# Transitions from Injection-Drug-Use-Concentrated to Self-Sustaining Heterosexual HIV Epidemics: Patterns in the International Data

**DOI:** 10.1371/journal.pone.0031227

**Published:** 2012-03-01

**Authors:** Don C. Des Jarlais, Jonathan P. Feelemyer, Shilpa N. Modi, Kamyar Arasteh, Bradley M. Mathers, Louisa Degenhardt, Holly Hagan

**Affiliations:** 1 The Baron Edmond de Rothschild Chemical Dependency Institute, Beth Israel Medical Center, New York, New York, United States of America; 2 National Drug and Alcohol Research Centre, University of New South Wales, Sydney, New South Wales, Australia; 3 College of Nursing, New York University, New York, New York, United States of America; McGill University AIDS Centre, Canada

## Abstract

**Background:**

Injecting drug use continues to be a primary driver of HIV epidemics in many parts of the world. Many people who inject drugs (PWID) are sexually active, so it is possible that high-seroprevalence HIV epidemics among PWID may initiate self-sustaining heterosexual transmission epidemics.

**Methods:**

Fourteen countries that had experienced high seroprevalence (<20%) HIV epidemics among PWID and had reliable data for injection drug use (IDU) and heterosexual cases of HIV or AIDS were identified. Graphs of newly reported HIV or AIDS cases among PWID and heterosexuals were constructed to identify temporal relationships between the two types of epidemics. The year in which newly reported cases among heterosexuals surpassed newly reported cases among PWID, aspects of the epidemic curves, and epidemic case histories were analyzed to assess whether it was “plausible” or “highly unlikely” that the HIV epidemic among PWID might have initiated the heterosexual epidemic in each country.

**Results:**

Transitions have occurred in 11 of the 14 countries. Two types of temporal relationships between IDU and heterosexual HIV epidemics were identified, rapid high incidence transitions vs. delayed, low incidence transitions. In six countries it appears “plausible” that the IDU epidemic initiated a heterosexual epidemic, and in five countries it appears “highly unlikely” that the IDU epidemic initiated a heterosexual epidemic. A rapid decline in incidence among PWID after the peak year of new cases and national income were the best predictors of the “highly unlikely” initiation of a heterosexual epidemic.

**Discussion:**

Transitions from IDU concentrated epidemics to heterosexual epidemics are common in countries with high seroprevalence among PWID though there are distinct types of transitions. Interventions to immediately reduce HIV incidence among PWID may reduce the likelihood that an IDU epidemic may initiate a heterosexual epidemic.

## Introduction

HIV can spread very rapidly among people who inject drugs (PWID) with incidence rates of 10% or more per year and prevalence reaching 30% to 60% within periods of five years [1], [2], [3], [4]. Most PWID are sexually active, and many do not consistently use condoms, so that sexual transmission of HIV from seropositive PWID to non-injecting sexual partners is extremely likely. One of the most important potential consequences of high seroprevalence HIV epidemics among PWID is that they may initiate self-sustaining epidemics among non-drug-injecting heterosexuals.

The potential transition from an HIV/AIDS epidemic dominated by injecting-related transmission to an epidemic dominated by heterosexual transmission has been most frequently considered in terms of potential “bridge populations”—subgroups that may be particularly important in a transition from injecting-related HIV transmission to heterosexual transmission. Female injectors who engage in commercial sex work and males injectors who utilize the services of non-injecting female sex workers are probably the two most commonly cited “bridge populations” [5], [6].

Studies of females injectors who engage in commercial sex work have been conducted in a moderately large number of areas, including Eastern Europe, South America and southeast Asia, with the percentage of females injectors who engage in commercial sex work ranging from 4% in France [7] to as high as 84% in the Netherlands [8]. Studies of males injectors who utilize services of female commercial sex workers have also been conducted in a number of areas, with the percentages of male injectors who utilize female commercial sex workers ranging from 6% in Ukraine [9] to as high as 81% in Brazil [10]. These studies of bridge populations clearly demonstrate the potential for high seroprevalence epidemics among PWID to lead to substantial heterosexual transmission of HIV.

Despite these studies of potential bridge populations, we do not have a systematic understanding of how high seroprevalence HIV epidemics among PWID may or may not initiate self-sustaining heterosexual transmission epidemics. Given that many PWID are sexually active and do not use condoms consistently, it is highly plausible that some transmission of HIV will occur between PWID and their non-drug injecting sexual partners. However, this sexual transmission might be relatively self-contained. If PWID do not have many sexual partners and if the non-injecting sexual partners of PWID do not themselves have many sexual partners, then the virus would be unlikely to spread beyond the sexual partners of PWID. While a high percentage of the sexual partners of PWID may become infected with HIV, the HIV epidemic among PWID would not initiate a self-sustaining heterosexual epidemic. In contrast, if HIV were to spread from PWID to a “bridge population” whose members themselves had large numbers of heterosexual partners, e.g., commercial sex workers, then the initial HIV epidemic among PWID may well be followed by self-sustaining heterosexual transmission.

Determining the conditions under which an HIV epidemic among PWID could lead to sexual transmission that is essentially contained among the immediate sexual partners of the PWID versus the initiating a self-sustaining heterosexual epidemic requires research that compares outcomes of high seroprevalence epidemics among PWID across geographic areas rather than of bridge populations in a single area.

In this report, we examine relationships among high seroprevalence IDU epidemics and heterosexual transmission epidemics in 14 countries in Asia, Europe, and South America, and assess the likelihood that the HIV epidemic among PWID might have initiated a heterosexual epidemic.

## Methods

### Countries with high HIV prevalence epidemics among PWID

We selected countries as the area for analysis of high HIV/AIDS prevalence epidemics among PWID. We recognize that there may be considerable variation in HIV infection among PWID in different parts of the same country, particularly for large, diverse countries. However, longitudinal data on newly reported HIV or AIDS cases among PWID and among non-drug-injecting heterosexuals was generally available only at the country level. This was particularly true for HIV/AIDS cases among PWID and non-injecting heterosexuals in low/middle income countries, which were of great interest. We defined high seroprevalence HIV epidemics among PWID as countries/areas in which HIV prevalence among PWID reached 20% or greater. These areas were identified from published papers by Mathers et al [11] and Aceijas et al [12]. The countries identified as having experienced 20% or greater HIV prevalence among PWID and were used in the main analysis were Argentina, Brazil, China, Estonia, France, Indonesia, Italy, the Netherlands, Russia, Scotland, Spain, Ukraine, and Vietnam. Four countries were excluded as a result of unreliable or incomplete HIV/AIDS surveillance: Kenya, Cambodia, Nepal and Burma/Myanmar. We conducted a supplementary analysis of Thailand because molecular epidemiology suggests that the initial HIV epidemic among PWID in Thailand was clade B, while the HIV epidemic among heterosexuals in that country was primarily clade E [13].

### Transitions between IDU concentrated epidemics and self-sustained heterosexual epidemics

UNAIDS and National Ministry of Health surveillance reports were used to plot annual newly-reported HIV or AIDS cases among PWID and heterosexuals by year for each country. We used newly-reported HIV cases for all countries for which this data were available for the history of the HIV/AIDS epidemic, and newly-reported AIDS cases for those countries for which HIV case data were not available for the history of the epidemic. Data were abstracted from the start of the epidemic in a specific country through the most recent data available (in most cases, 2008). Key contacts at UNGASS, UNAIDS, and country health departments were contacted to obtain additional information on newly reported HIV/AIDS cases among PWID and heterosexuals.

We defined the “transition point” between an injection drug use (IDU) epidemic and a heterosexual epidemic as the time when newly reported cases among non-drug injecting heterosexuals first surpassed newly reported cases among PWID. For almost all countries in which heterosexual cases surpassed cases among PWID, it was possible to identify a single year for the transition.

Different types of transitions were identified by examining the relationship between the peak year for newly reported HIV or AIDS cases among PWID, the year in which newly reported cases among heterosexuals surpassed newly reported cases among PWID, and trends in heterosexual cases during the 3 to 4 year period around the time at which new heterosexual cases surpassed new cases among PWID.

### Identifying Factors Associated with different types of transitions between IDU and heterosexual epidemics

We analyzed aspects of the curve for newly reported cases among PWID, and conducted systematic literature searches, contacted public health authorities and experts in the field to identify possible characteristics of heterosexual cases of HIV/AIDS that were reported near the time of the transition.

The factors we examined included:

1. General Context: High versus low/middle national income countries
2. Factors associated with HIV transmission among PWID:
  - Rapidity of the rise to peak HIV/AIDS cases among PWID, with a “very rapid rise” defined as an increase of >500% in the 5 year span prior to the peak of newly reported cases among PWID. The data displayed in figures S1, S2, S3, and S4 were used to calculate this factor.
  - Persistence of a high rate of newly reported HIV/AIDS cases among PWID, with persistence (defined as <30% reduction in the number of newly reported cases in the 3 year span after peak or during the transition period of newly reported cases among PWID) versus a rapid decline in newly reported cases among PWID (defined as >30% reduction in newly reported cases in the 3 year span after peak or during the transition period of newly reported cases among PWID). The data displayed for figures S1, S2, S3, and S4 were used to calculate this factor.
  - HIV prevention program implementation dates for PWID
3. Factors potentially associated with sexual transmission from PWID to non-drug injecting heterosexuals:
  - Participation in commercial sex work among females who inject drugs,
  - Use of commercial sex workers by males who inject drugs,
  - Syphilis rates among PWID.
4. Special characteristics of non-drug injecting heterosexuals with HIV/AIDS around the time when heterosexual cases exceeded cases among PWID:
  - Whether heterosexual transmission cases were believed to be occurring outside of the country,
  - Whether heterosexual cases were reported among immigrants to the country,
  - Syphilis rates among the general population.

We used this information to construct brief case histories of the HIV epidemics among PWID and heterosexuals in each country.

### Data Analysis

We examined potential transitions in all countries that experienced high HIV prevalence (20% or higher at a national level) among PWID and had what we considered to be reliable data. We were able to utilize data from 14 of the 18 countries for a sampling fraction of 78%. Visual inspection and quantification of the trends in newly reported HIV/AIDS cases among PWID and non-injecting heterosexuals were used to identify patterns in the transitions between HIV epidemics among PWID and among non-injecting heterosexuals in each country.

A qualitative assessment of the likelihood that the HIV epidemic among PWID initiated the heterosexual epidemic was made based on the type of transition and the case history for each country.

## Results

Figures S1, S2, S3, and S4 show newly reported HIV or AIDS cases by year for PWID and non-drug-injecting heterosexuals for the countries in this analysis. Transition points—where newly reported cases among heterosexuals surpassed newly reported cases among PWID—occurred in 11 of the 13 areas studied. Three patterns occurred in the transitions:

1. Countries that have not experienced a transition. In these countries the number of newly reported cases among PWID continued to exceed the number of newly reported cases among heterosexuals through the end the most recent data available for each country. Transitions had not yet occurred in Estonia, Russia and Vietnam. (Estonia moved to a surveillance system that identified new HIV cases only by gender and not by transmission risk).
2. Pattern A transitions. These transitions occurred when: a) new cases among PWID were at a high level (>80% of the peak of newly reported cases among PWID), b) within 2 years of the peak annual number of newly reported cases among PWID, and c) when newly reported cases among heterosexuals were increasing (at least a 25% increase of newly reported cases among heterosexuals in the 3 to 4 year period around the transition). Countries that experienced Pattern A transitions included: Argentina, Brazil, China, Indonesia, the Netherlands, and Ukraine.
3. Pattern B transitions. These transitions occurred: a) six to ten years after the peak of newly reported cases among PWID, b) at a rate <50% of the peak of newly reported cases among PWID, and c) when newly reported cases among heterosexuals were stable or declining (no more than a 30% increase in new heterosexual cases in the four year period around the transition). Countries that experienced Pattern B transitions included: Italy, Scotland and Spain.

France was the one country that had experienced a transition but did not clearly fit into the Pattern A versus Pattern B distinction. The transition in France occurred 2 years after the peak of newly reported cases among PWID, and at a rate of 86% of the peak year for newly reported cases among PWID (1993), but at a time when newly reported cases among heterosexuals were clearly declining and not increasing (there was a 24.3% decline in newly reported cases among heterosexuals in the four year period around the transition).

Thailand's HIV graph is also presented in Figure S4; further assessment of the epidemics in Thailand is given in the case histories noted below.

There were no “reversals” of changes in the relative numbers of newly reported HIV or AIDS cases among PWID and heterosexuals. That is, there were no countries in which newly reported cases among heterosexuals exceeded newly reported cases among PWID and then newly reported cases among PWID later exceeded newly reported cases among heterosexuals. Once cases among heterosexuals exceeded cases among PWID, this appeared to be a permanent change in the HIV epidemics in these countries.

### Case histories of countries where transitions have occurred

1) Countries with Pattern A transitions:

Argentina experienced a rapid increase in AIDS cases among PWID, with an increase of 275% for the five year span preceding the peak year of newly reported AIDS cases in 1996. This was followed by a persistent high rate of newly reported cases with only an 18% decrease in newly reported AIDS cases in the three year span after the peak year. HIV prevention programs for PWID were begun in 1995 and scaled up in the late 1990's [14]. In 1995–1996, when PWID registered the highest number of AIDS cases, 41% of total AIDS cases were attributed to IDU transmission [15]. One study in Grand Rosario reported that 19% of female injectors participated in commercial sex work in the early 2000's [16].

Brazil experienced a rapid increase in AIDS cases among PWID, with an increase of 162% for the three year span preceding the peak year of newly reported AIDS cases in 1995. This was followed by a persistent high rate of newly reported AIDS cases with only a very slight (1%) decrease in newly reported cases in the three year span after the peak year. HIV prevention programs for PWID were begun in 1989 but not scaled up until the mid 1990's [17]. In 1995, PWID registered the highest number of AIDS cases which represented 22% of total AIDS cases. A high number of female injectors engage in commercial sex work ranging from 25% to 53% [18], [19]. One study in Sao Paulo reported that 81% of male injectors of whom 65% were HIV positive, admitted to utilizing commercial sex workers [10].

China experienced a rapid increase in HIV cases among PWID, with an increase of 188% for the five year span preceding the peak year of newly reported HIV cases in 2007. This was followed by a persistent high rate of newly reported HIV cases among PWID, with only a 21% decrease in newly reported cases in the three year span after the peak year. HIV prevention programs for PWID were begun in 1999 and scaled up between 2005 and 2008 [20], [21]. A high percentage of female injectors engage in commercial sex work in China; a systematic review reported that 52% to 98% of female injectors engaged in commercial sex work [22]. Among male injectors, several studies in Sichuan, Yunnan, and Dazhou have found that the percentage of male injectors that utilize commercial sex workers ranged from 19% to 34% [23], [24], [25], [26].

Indonesia experienced a rapid increase in HIV cases among PWID, with an increase of 2433% for the five year span preceding the peak year of newly reported HIV cases in 2006. This was followed by a persistent high rate of newly reported HIV cases with a 24% decrease in newly reported cases in the three year span after the peak year. HIV prevention programs for PWID were begun in 2003 and scaled up between 2003 and 2009 [27]. In 2006, when PWID registered the highest number of HIV cases, 53% of total HIV cases were attributed to IDU transmission [28]. A national study in Indonesia conducted in 2007 reported that 19% of female injectors participated in commercial sex work [29]. A review of eight primary studies throughout Indonesia between 2004 and 2006 reported moderate to high levels of male injectors utilizing commercial sex workers, ranging from 21% to 79% [29], [30]. A national survey of male injectors in Indonesia conducted in 2004 reported that approximately 40% throughout the country had utilized commercial sex workers, with half of those cities reporting rates above 50%. Additionally, in Indonesia, there is a large group of heterosexual male injectors who utilize transgender sex workers or “waria” with studies reporting up to 80% of men in heterosexual partnerships utilizing these services [29].

The Netherlands experienced a rapid increase in AIDS cases among PWID, with an increase of 76% for the five year span preceding the peak year of newly reported AIDS cases in 1995. The transition, where new heterosexual cases surpassed new IDU cases, occurred during this period. HIV prevention programs for PWID were begun in 1984 and scaled up in late 1980s and early 1990s [31]. In 1995, when PWID registered the highest number of AIDS cases, 14% of total AIDS cases were attributed to IDU transmission; this peak occurred after heterosexual transmission of AIDS cases exceeded the number of AIDS cases among PWID [32]. As the transition occurred prior to the peak of IDU cases, “persistence of high incidence” among PWID was assessed for the 3 years around the transition in the Netherlands. There is a high prevalence of female injectors in the Netherlands; among seventeen studies with PWID samples, 32% to 55% of the PWID were female. Additionally, among female injectors, the prevalence of commercial sex work is very high, with two studies reporting commercial sex rates between 83% and 84% [8], [33].

Ukraine experienced a rapid increase in HIV cases among PWID, with an increase of 62.7% for the two year span preceding the peak year of newly reported HIV cases in 1997. This was followed by persistent high rate of new cases among PWID, with a moderate decline over the next several years but then an increase in newly reported cases in later years. HIV prevention programs for PWID were begun in 2004 and scaled up between 2004 and 2007 including expansion of needle exchange services [34]. In 1997, when PWID registered the highest number of HIV cases, 84% of total HIV cases were attributed to IDU transmission [35]. Three studies report a moderate level of female injectors who are also commercial sex workers, ranging from 7% to 25% [9], [18]. One study in Ukraine found low levels of male injectors utilizing commercial sex workers, reporting that 7% admitted to utilizing commercial sex workers at one point in their injecting career [9].

2) Countries with Pattern B transitions.

There were three countries in which the cases among heterosexuals exceeded cases among PWID after very substantial declines in the rate of new cases among PWID—of at least 50% and typically 6 to 10 years after peak number of new cases among PWID.

In Italy, harm reduction services, particularly opioid substitution therapy, were implemented on a large scale in the late 1980s. Newly reported cases of AIDS among PWID peaked in 1994, and then declined rapidly through 1999. Heterosexual AIDS cases exceeded AIDS cases among PWID between 2000 and 2001. At this time, syphilis was increasing in the general population and the percentage of AIDS cases among migrants was increasing, from 3% in 1993 to nearly 21% in 2007, with the great majority of the cases among migrants attributed to heterosexual transmission [36]

In Scotland, HIV prevention programs for PWID, including syringe exchange and methadone maintenance, were rapidly scaled up in the mid- to late-1980s and AIDS cases among PWID declined rapidly. Heterosexual transmission was primarily associated with travel to other countries. An analysis of cumulative heterosexual cases indicated that 75% were probably acquired in Africa, 6% in Asia, and less than 20% in Scotland [37].

In Spain, newly reported AIDS cases among PWID peaked in 1994, and then declined rapidly. Important factors in the decline were a change in route of drug administration from injecting to non-injecting drug use [38] and the implementation of harm reduction services [39]. Spain had a large number of commercial sex workers (estimated at 500,000) [40], and high rates of syphilis among commercial sex workers [40], [41]. The commercial sex workers, however, were primarily immigrants from Latin America, Eastern Europe, and Africa, and not PWID [40].

### France

France experienced a rapid increase in AIDS cases among PWID, with an increase of 134% for the five year span preceding the peak year of newly reported AIDS cases in 1993. The high rate of new cases among PWID did not persist, however, as there was a 35% decrease in newly reported cases in the three year span after the peak year. HIV prevention programs for PWID were begun in 1995 and scaled up in late 1990's [42]. One study in Paris reported that only 8% of female injectors participated in commercial sex work in the early 2000's [43]. Heterosexual transmission in France is highly linked to foreign migrants. In 2003, 69% of HIV diagnoses among heterosexuals were attributed to migrants of whom 65% were women [44]. Specifically, many of the migrants were of sub-Saharan African origins; between 2003 and 2006, 33% of newly reported AIDS cases were among migrants from sub-Saharan African countries, and were predominately infected through heterosexual sex [45].

### Thailand: Clade Analysis of Newly Reported HIV Cases

As noted above, we did not include Thailand in the above analyses because of molecular epidemiology data indicating that the HIV epidemic among PWID was a different HIV clade than the heterosexual epidemic [13]. We did, however, examine the epidemic curves for HIV cases among PWID and heterosexuals in Thailand. HIV incidence among PWID in Thailand was characterized by both a rapid rise and a rapid fall in reported AIDS cases among PWID. Newly reported cases increased rapidly from 522 cases in 1986 to 33,000 cases in 1988, but then fell very rapidly, to 7,574 cases in 1990, 5,341 cases in 1995, 2,794 in 2000, and 1100 cases in 2004 [46]. Thus, Thailand did not experience a persistent high rate of new cases among PWID. The point where newly reported heterosexual HIV cases surpassed cases among PWID occurred in 1991, two years after the peak of newly reported HIV cases among PWID. Figure S4 contains a graph of cases among PWID and heterosexuals over time, based on national surveillance data.

### Qualitative summary analysis

The research presented here is observational epidemiology, and as such we did not have the opportunity to experimentally alter any of the conditions among people who inject drugs or non-drug injecting heterosexuals in any of these countries. Nevertheless, we do believe that the data provide relatively strong support for some conclusions about the possible conditions under which HIV epidemics among PWID are or are not likely to initiate self-sustained HIV epidemics among non-drug injecting heterosexuals.

Based on the types of transitions (Pattern A vs. Pattern B) and the information on characteristics of the heterosexual cases near the time of the transition we estimated the likelihood that the HIV epidemic among PWID was an important factor in the initiation of the heterosexual transmission epidemic in each of the countries. We used a dichotomous classification: either it was “highly unlikely” that the IDU epidemic initiated a heterosexual transmission in the country, or it was “plausible” that the IDU epidemic initiated a heterosexual transmission in the country.

Pattern B transitions occurred in Italy, Scotland and Spain, where cases among PWID had declined substantially (at least 50% from peak) and with cases among heterosexuals surpassing new cases among PWID well after (6 to 10 years) the peak number of new cases among PWID, and when new cases among heterosexuals were relatively stable. The characteristics of the heterosexual transmission cases differed from the characteristics of the cases among PWID in France, Italy, Scotland, Spain and Thailand (where heterosexual cases were infected with a different clade of HIV). These characteristics would suggest that it was “highly unlikely” that the epidemics among PWID initiated the heterosexual transmission epidemics in France, Italy, Scotland, Spain, or Thailand. Sexual transmission from PWID to sexual partners undoubtedly occurred, but it appears that the heterosexual epidemics occurred independently of the IDU epidemics in these countries.

In contrast, the Pattern A transitions in Argentina, Brazil, China, France, Indonesia, and Ukraine occurred close in time to the peak of new cases among PWID, at a high level of new cases among PWID, and without known epidemiologic separation of the IDU and heterosexual transmission cases. Additionally new heterosexual cases were increasing during the 3 to 4 years around the transition. This clearly does not prove that the IDU epidemics initiated the heterosexual epidemics in these countries. Rather, we would conclude it remains “plausible” that the IDU epidemics in these countries were important factors in the initiation of the heterosexual epidemics.

Table 1 summarizes the type of transition, information about special characteristics of the heterosexual cases, persistence of high rates of new cases among PWID, and our assessment of the likelihood that the HIV epidemic was an important factor in initiating the heterosexual epidemic in the 11 countries in which transitions have occurred (including the supplemental analysis for Thailand).

**Table 1:** Special Characteristics and Transition Types by Country.

Country	Type of Transition	Special Characteristics of Heterosexuals*	Persistence of High Rate of New Cases among PWID**	Qualitative Assessments of Initiating Heterosexual Epidemic
Argentina	Pattern A		Yes	Plausible
Brazil	Pattern A		Yes	Plausible
China	Pattern A		Yes	Plausible
Indonesia	Pattern A		Yes	Plausible
Netherlands	Pattern A		Yes	Plausible
Ukraine	Pattern A		Yes	Plausible
Italy	Pattern B	Migrant Populations	No	Highly Unlikely
Scotland	Pattern B	Infected Abroad	No	Highly Unlikely
Spain	Pattern B	Many infected via sex with commercial sex workers (Majority of CSW were immigrants and only few were PWID)	No	Highly Unlikely
France	Mixed	Migrant Populations	No	Highly Unlikely
Thailand	Mixed	HIV epidemic among PWID predominantly clade B, while the HIV epidemic among heterosexuals was primarily clade E	No	Highly Unlikely

*From literature search.

**Persistence is operationally defined as less than a 30% decline in new IDU cases around the transition period or over the 3 year span after the peak in IDU cases.

## Discussion

To our knowledge, this is the first multinational study of relationships between IDU and heterosexual HIV/AIDS epidemics. While studies of individual HIV epidemics are clearly important, comparative analyses provide potential insights about the circumstances under which IDU epidemics may initiate heterosexual epidemics.

There were two characteristics that distinguished the “highly unlikely” from the “plausible” linkages between the IDU and heterosexual epidemics in the 11 countries that have experienced transitions. First, the “highly unlikely” cases occurred primarily in high-income countries (4 out of 5 of the “highly unlikely” cases were in high income countries versus only 1 out of 6 of the “plausible” cases). This relationship may reflect a lack of resources for addressing HIV transmission in low/middle income countries and/or possibly greater stigmatization of populations such as PWID in many low/middle income countries.

The factor that most clearly distinguished the “highly unlikely” versus the “plausible” cases, however, was the persistence of a high rate of new HIV/AIDS cases among PWID (operationally defined as less than a 30% decline in new cases in the three year span after the peak year). Persistent high rates of new cases among PWID occurred in all 6 of the countries in which it was “plausible” that the IDU epidemic initiated the heterosexual epidemic and in none of the 5 countries in which it was “highly unlikely” that the IDU epidemic initiated the heterosexual epidemic. Persistent high rates of new cases among PWID might contribute to sexual transmission of HIV to persons who do not inject through several mechanisms. Persistent high rates of new HIV/AIDS cases among PWID could reflect continuation of high rates of injecting and sexual risk behaviors. Persistent high rates of new cases might also reflect large numbers of new initiates into drug injection. New injectors would be likely to engage in high rates of injecting and sexual risk behaviors [47]. Additionally, new injectors could replenish the group of very high-risk susceptibles—in contrast to potential saturation of very high-risk susceptibles in a PWID population that was not increasing. Persistent high rates of new infections among PWID may reflect law enforcement practices that restrict PWID from obtaining sterile injecting equipment and protecting themselves by reducing sharing behaviors [48], [49]. Perhaps most importantly, the persistence of high rates of newly reported cases among PWID would include large numbers of acute HIV infections over an extended time period. HIV infectiousness is particularly high during acute infection [50], so that continuing high rates of new infections among PWID would increase transmission to both other PWID and to non-drug-injecting sexual partners.

### Limitations

This study has a number of limitations that should be considered. It was not possible to use HIV data among PWID and heterosexuals for all areas. Rather we had to use newly reported AIDS cases for countries that experienced early epidemics, before HIV cases became the standard for reporting. We necessarily relied upon official public health reporting of the data, and there undoubtedly is considerable imprecision as to when new cases occurred and when they were reported. We also had to aggregate data to a national level, even though HIV epidemics are probably best analyzed at a local level. Data were typically not available for local areas in low/middle income countries and we were particularly interested in the relationships between IDU and heterosexual epidemics in those countries. National HIV/AIDS data is frequently used in UNAIDS/WHO reporting [14], [27], [51], [52], planning for AIDS prevention and treatment, e.g., the US National HIV/AIDS Strategy [53], and in funding decisions made by international donors. Given the widespread use of national HIV/AIDS data, it seemed appropriate to analyze data at this level. Finally, we were not able to include consideration of HIV/AIDS among men who have sex with men (MSM) in the different countries. MSM who also have sex with women are another possible bridge to sustained heterosexual transmission epidemics. The majority of countries with high rate/rapid transitions, however, initially had HIV epidemics driven by injecting drug use, with MSM epidemics that followed later [54].

These limitations would introduce uncertainty regarding any specific data point—including newly reported cases in a given year. Nevertheless, it is extremely difficult to see how these limitations would have produced the patterns observed in this report. It is particularly difficult to envision how these limitations would have led to erroneous classification of the Pattern A versus Pattern B transitions. It is also difficult to see how these limitations would have produced the conclusions that it is “highly unlikely” that HIV among PWID was an important factor in the initiation of heterosexual transmission in Italy, Scotland and Spain.

### Implications for further research

As a first international comparison of the relationships between HIV epidemics among PWID and among heterosexuals, this study should be seen as hypotheses generating rather than hypothesis testing. The most important hypothesis from the data presented here concerns persistent high rates of new cases among PWID. Persistent high rates of new cases among PWID did not occur in any of the countries we assessed as “highly unlikely” that the IDU epidemic initiated the heterosexual epidemic. Persistent high rates of new cases among PWID did occur in all of the countries we assessed as “plausible” that the IDU epidemic was important in initiating the heterosexual epidemic. Thus, these data would suggest that persistent high rate of new cases among PWID (less than a 30% decline in new cases in the three year span after the peak year of cases among PWID) is a necessary condition for an HIV epidemic among PWID to be important in initiating an HIV epidemic among heterosexuals. This could be tested by examining relationships between IDU and heterosexual HIV epidemics at smaller geographic units, such as cities.

Even if persistent high rates of new cases among PWID is a necessary condition, there may be additional important factors. Closer analysis of the “highly unlikely” cases might identify additional factors that kept HIV infection among PWID from initiating a heterosexual epidemic. In the countries where we assessed a “plausible” relationship between the IDU epidemic and the initiation of the heterosexual epidemic, it would be useful to examine factors such as sexual mixing patterns among PWID and non-drug injecting heterosexuals through “bridge populations” (female injectors who engage in commercial sex work, male injectors that utilize the services of commercial sex workers) and the prevalence of sexually transmitted diseases such as syphilis that would biologically facilitate HIV transmission. It would seem very likely that there would be multiple factors important in initiating a heterosexual epidemic, not just persistent high rates of new cases among PWID.

### Public health implications

The most appropriate form for testing the hypothesis of the importance of persistent high rates of new cases among PWID would be in the form of public health actions: Do interventions that prevent “persistent high rates of new cases” among PWID decrease the likelihood that a high seroprevalence HIV epidemic among PWID will be closely followed by an epidemic among heterosexuals? There are a number of evidence-based interventions (needle/syringe programs, medication assisted treatment for drug dependence, anti-retroviral treatment) that can reduce HIV transmission in high prevalence settings. In particular, there is considerable evidence that large scale needle/syringe access programs and drug abuse treatment programs can reduce HIV transmission among PWID in relatively short periods of time [39], [55], [56], [57], [58], [59]. The public health challenge would be to scale up the interventions as rapidly as feasible, and the research challenge would be to carefully monitor HIV transmission to non-injecting sexual partners of PWID.

A final comment concerns the context of interventions to reduce the likelihood of high rate/rapid transitions from IDU concentrated to sustained heterosexual epidemics. All of the potential interventions noted above involve health workers working in collaboration with PWID, their sexual partners and persons at high risk for beginning to inject drugs. Actions that stigmatize PWID or make it difficult for them and their sexual partners to participate in HIV prevention programs may be counterproductive and increase the chances of high incidence HIV/AIDS epidemics among non-drug-injecting heterosexuals.

## Supporting Information

Figure S1 **No Transition Countries.** Locations that had not experienced a transition from IDU-concentrated to heterosexual sustained HIV/AIDS epidemics, and newly reported HIV/AIDS cases among PWID still surpass newly reported heterosexual HIV/AIDS cases. Each data point corresponds to the number of newly reported HIV/AIDS cases, by risk group, for each year data was available.
(TIF)

Figure S2 **Pattern A transitions from IDU-concentrated to heterosexual HIV epidemics.** Locations that experienced transitions from IDU-concentrated to heterosexual sustained HIV/AIDS epidemics at the peak newly reported HIV/AIDS cases among PWID. Each data point corresponds to the number of newly reported HIV/AIDS cases, by risk group, for each year data was available.
(TIF)

Figure S3 **Pattern B transitions from IDU-concentrated to heterosexual epidemics.** Locations that experienced transitions from IDU-concentrated to heterosexual sustained HIV/AIDS epidemics and low levels of newly reported HIV/AIDS cases among PWID. Each data point corresponds to the number of newly reported HIV/AIDS cases, by risk group, for each year that surveillance data was available.
(TIF)

Figure S4 **Mixed Transition from IDU-concentrated to heterosexual epidemics.** Locations that had experienced a transition from IDU-concentrated to heterosexual sustained HIV/AIDS epidemics, and newly reported HIV/AIDS cases among PWID still surpass newly reported heterosexual HIV/AIDS cases. Each data point corresponds to the number of newly reported HIV/AIDS cases, by risk group, for each year that surveillance data was available. **These countries were separated into a separate category due to their unique transitions that did not fit the trends of pattern A or pattern B transitions.**
(TIF)

## References

[1] Health AOCIo (2009).

[2] HPS (2009). Focus: HIV in Scotland 2008 Review.

[3] UNAIDS (2010). UNAIDS Report on the global AIDS epidemic 2010.

[4] Veirmer A (2008). HIV In Estonia..

[5] Liu H, Grusky O, Li X, Ma E (2006). Drug users: a potentially important bridge population in the transmission of sexually transmitted diseases, including AIDS, in China.. Sex Transm Dis.

[6] Zhu TF, Wang CH, Lin P, He N (2005). High risk populations and HIV-1 infection in China.. Cell Res.

[7] Aim G, De Vincenzi I, Ancelle-Park R, Brunet JB, Catalan F (1989). HIV infection in French prostitutes.. AIDS.

[8] van den Hoek A, van Haastrecht HJ, Coutinho RA (1990). Heterosexual behaviour of intravenous drug users in Amsterdam: implications for the AIDS epidemic.. AIDS.

[9] Balakireva O, Teltschik A, Bondar T, Sereda Y, Sakovych O (2010). Social and Behavioral Research as a Basis for Implementing Effective Methods of HIV Prevention among Most-at-Risk Adolescents..

[10] Dunn J, Laranjeira RR (2000). HIV-risk behaviour among non-heroin using cocaine injectors and non-injectors in Sao Paulo, Brazil.. AIDS Care.

[11] Mathers BM, Degenhardt L, Phillips B, Wiessing L, Hickman M (2008). Global epidemiology of injecting drug use and HIV among people who inject drugs: a systematic review.. Lancet.

[12] Aceijas C, Stimson GV, Hickman M, Rhodes T (2004). Global overview of injecting drug use and HIV infection among injecting drug users.. AIDS.

[13] Tovanabutra S, Watanaveeradej V, Viputtikul K, De Souza M, Razak MH (2003). A new circulating recombinant form, CRF15_01B, reinforces the linkage between IDU and heterosexual epidemics in Thailand.. AIDS Res Hum Retroviruses.

[14] UNAIDS (2010).

[15] Ministry of Health of the Nation (2010).

[16] Multare S, Mazzetti MC, Shinzato R, Macias J, Weissenbacher M (1992). HIV prevalence among female prostitutes in Buenos Aires (Argentina).. Int Conf AIDS.

[17] Mesquita F, Kral A, Reingold A, Bueno R, Trigueiros D (2001). Trends of HIV infection among injection drug users in Brazil in the 1990s: the impact of changes in patterns of drug use.. J Acquir Immune Defic Syndr.

[18] Cleland CM, Des Jarlais DC, Perlis TE, Stimson G, Poznyak V (2007). HIV risk behaviors among female IDUs in developing and transitional countries.. BMC Public Health.

[19] de Carvalho HB, Mesquita F, Massad E, Bueno RC, Lopes GT (1996). HIV and infections of similar transmission patterns in a drug injectors community of Santos, Brazil.. J Acquir Immune Defic Syndr Hum Retrovirol.

[20] Mathers BM, Degenhardt L, Ali H, Wiessing L, Hickman M (2010). HIV prevention, treatment, and care services for people who inject drugs: a systematic review of global, regional, and national coverage.. Lancet.

[21] Liu B, Sullivan SG, Wu Z (2007). An evaluation of needle exchange programmes in China.. AIDS.

[22] Yang X (2005). Does where we live matter? Community characteristics and HIV and sexually transmitted disease prevalence in southwestern China.. Int J STD AIDS.

[23] Lau JT, Tsui HY, Zhang Y, Cheng F, Zhang L (2008). Comparing HIV-related syringe-sharing behaviors among female IDU engaging versus not engaging in commercial sex.. Drug Alcohol Depend.

[24] Lau JT, Zhang J, Zhang L, Wang N, Cheng F (2007). Comparing prevalence of condom use among 15,379 female sex workers injecting or not injecting drugs in China.. Sex Transm Dis.

[25] Gu J, Wang R, Chen H, Lau JT, Zhang L (2009). Prevalence of needle sharing, commercial sex behaviors and associated factors in Chinese male and female injecting drug user populations.. AIDS Care.

[26] Yao Y, Wang N, Chu J, Ding G, Jin X (2009). Sexual behavior and risks for HIV infection and transmission among male injecting drug users in Yunnan, China.. Int J Infect Dis.

[27] UNAIDS (2010).

[28] Directorate General CDC & EH Ministry of Health (2009).

[29] Heriawan R (2007). Behavioral Surveillance Survey (BSS) Result in Indonesia 2004–2005..

[30] Pisani E, Dadun, Sucahya PK, Kamil O, Jazan S (2003). Sexual behavior among injection drug users in 3 indonesian cities carries a high potential for HIV spread to noninjectors.. J Acquir Immune Defic Syndr.

[31] Witteveen E, Schippers G (2006). Needle and syringe exchange programs in Amsterdam.. Subst Use Misuse.

[32] Koedijk FDH, Vriend HJ, van Veen MG, Op de Coul ELM, van den Broek IVF, van Sighem AI, Verheij RA, van der Sande MAB (2008). Sexually transmitted infections, including HIV, in the Netherlands in 2008..

[33] van den Berg CH, Smit C, Bakker M, Geskus RB, Berkhout B (2007). Major decline of hepatitis C virus incidence rate over two decades in a cohort of drug users.. Eur J Epidemiol.

[34] MOH Ukraine (2009). Ukraine: National Report on Monitoring Progress Towards the ANGASS Declaration of Commitment on HIV/AIDS..

[35] MoH Ukraine (2008). Ukraine: National Report on Monitoring Progress Towards the UNGASS Declaration of Commitment on HIV/AIDS..

[36] Pezzoli MC, Hamad IE, Scarcella C, Vassallo F, Speziani F (2009). HIV infection among illegal migrants, Italy, 2004–2007.. Emerg Infect Dis.

[37] Yirrell DL, Goldberg DJ, Whitelaw J, McSharry C, Raeside F (1999). Viral subtype and heterosexual acquisition of HIV infections diagnosed in Scotland.. Sex Transm Infect.

[38] Barrio G, De la Fuente L, Royuela L, Diaz A, Rodriguez-Artalejo F (1998). Cocaine use among heroin users in Spain: the diffusion of crack and cocaine smoking. Spanish Group for the Study on the Route of Administration of Drugs.. J Epidemiol Community Health.

[39] Ramirez-Jonville A (2006). [Drug addiction: harm reduction policies in France and Spain].. Presse Med.

[40] Tarvainen s (2007). Spain divided over semi-legal prostitution..

[41] Estebanez P, Sarasqueta C, Fitch K, Zunzunegui V, Contreras G (1992). [Prevalence of HIV-1 and other sexually transmitted diseases among Spanish prostitutes].. Med Clin (Barc).

[42] Cook C (2010). The Global State of Harm Reduction 2010 Key issues for broadening the response.

[43] Gollub EL, Rey D, Obadia Y, Moatti JP (1998). Gender differences in risk behaviors among HIV+ persons with an IDU history. The link between partner characteristics and women's higher drug-sex risks. The Manif 2000 Study Group.. Sex Transm Dis.

[44] Lot F, Semaille C, Cazein F, Barin F, Pinget R (2004). Preliminary results from the new HIV surveillance system in France.. Euro Surveill.

[45] Semaille C, Cazein F, Pillonel J, Lot F, Le Vu S (2008). Four years of surveillance of recent HIV infections at country level, France, mid 2003–2006: experience and perspectives.. Euro Surveill.

[46] Ministry of Public Health Thailand (2009).

[47] Buchanan D, Tooze JA, Shaw S, Kinzly M, Heimer R (2006). Demographic, HIV risk behavior, and health status characteristics of “crack” cocaine injectors compared to other injection drug users in three New England cities.. Drug Alcohol Depend.

[48] Rhodes T, Platt L, Sarang A, Vlasov A, Mikhailova L (2006). Street policing, injecting drug use and harm reduction in a Russian city: a qualitative study of police perspectives.. J Urban Health.

[49] Booth RE, Lehman WE, Dvoryak S, Brewster JT, Sinitsyna L (2009). Interventions with injection drug users in Ukraine.. Addiction.

[50] Sagar M (2010). HIV-1 transmission biology: selection and characteristics of infecting viruses.. J Infect Dis.

[51] UNAIDS (2010).

[52] UNAIDS (2010).

[53] The White House Office of National AIDS Policy (2010). NATIONAL HIV/AIDS STRATEGY FOR THE UNITED STATES.

[54] Beyrer C, Baral SD, Walker D, Wirtz AL, Johns B (2010). The expanding epidemics of HIV type 1 among men who have sex with men in low- and middle-income countries: diversity and consistency.. Epidemiol Rev.

[55] Hope VD, Judd A, Hickman M, Sutton A, Stimson GV (2005). HIV prevalence among injecting drug users in England and Wales 1990 to 2003: evidence for increased transmission in recent years.. AIDS.

[56] Des Jarlais DC, Hagan H, Friedman SR, Friedmann P, Goldberg D (1995). Maintaining low HIV seroprevalence in populations of injecting drug users.. JAMA.

[57] Des Jarlais DC, Perlis T, Arasteh K, Torian LV, Beatrice S (2005). HIV incidence among injection drug users in New York City, 1990 to 2002: use of serologic test algorithm to assess expansion of HIV prevention services.. Am J Public Health.

[58] Carrieri MP, Spire B (2008). Harm reduction and control of HIV in IDUs in France.. Lancet.

[59] Kerr T, Small W, Buchner C, Zhang R, Li K (2010). Syringe sharing and HIV incidence among injection drug users and increased access to sterile syringes.. Am J Public Health.

