# Supplementary figures and images for: Transitions from Injection-Drug-Use-Concentrated to Self-Sustaining Heterosexual HIV Epidemics: Patterns in the International Data
Source: PLoS One. 2012 Mar 1;7(3):e31227. doi: 10.1371/journal.pone.0031227 (PMC3291614; doi:10.1371/journal.pone.0031227)

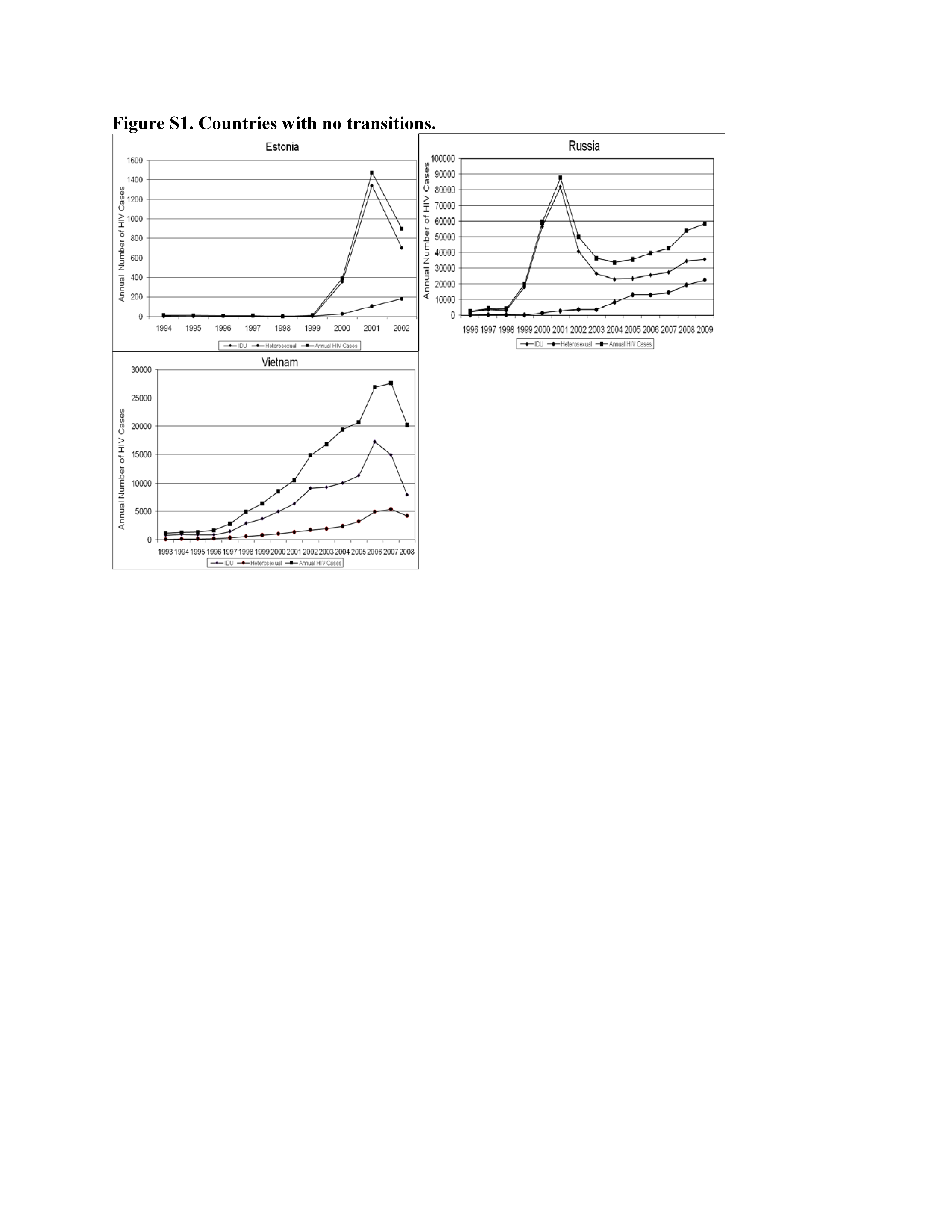

Supplement: Figure S1 — No Transition Countries. Locations that had not experienced a transition from IDU-concentrated to heterosexual sustained HIV/AIDS epidemics, and newly reported HIV/AIDS cases among PWID still surpass newly reported heterosexual HIV/AIDS cases. Each data point corresponds to the number of newly reported HIV/AIDS cases, by risk group, for each year data was available. (TIF) [file pone.0031227.s001.tif]

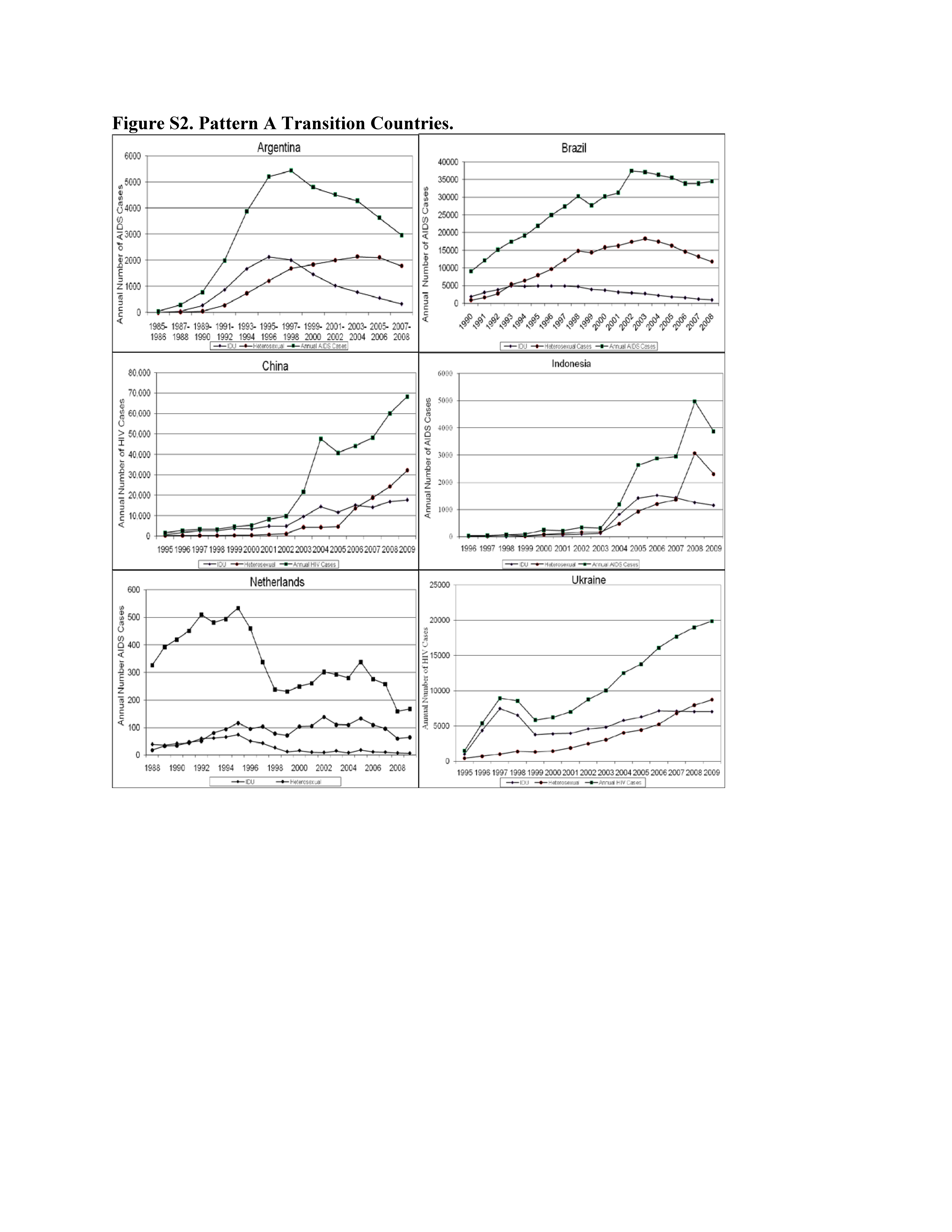

Supplement: Figure S2 — Pattern A transitions from IDU-concentrated to heterosexual HIV epidemics. Locations that experienced transitions from IDU-concentrated to heterosexual sustained HIV/AIDS epidemics at the peak newly reported HIV/AIDS cases among PWID. Each data point corresponds to the number of newly reported HIV/AIDS cases, by risk group, for each year data was available. (TIF) [file pone.0031227.s002.tif]

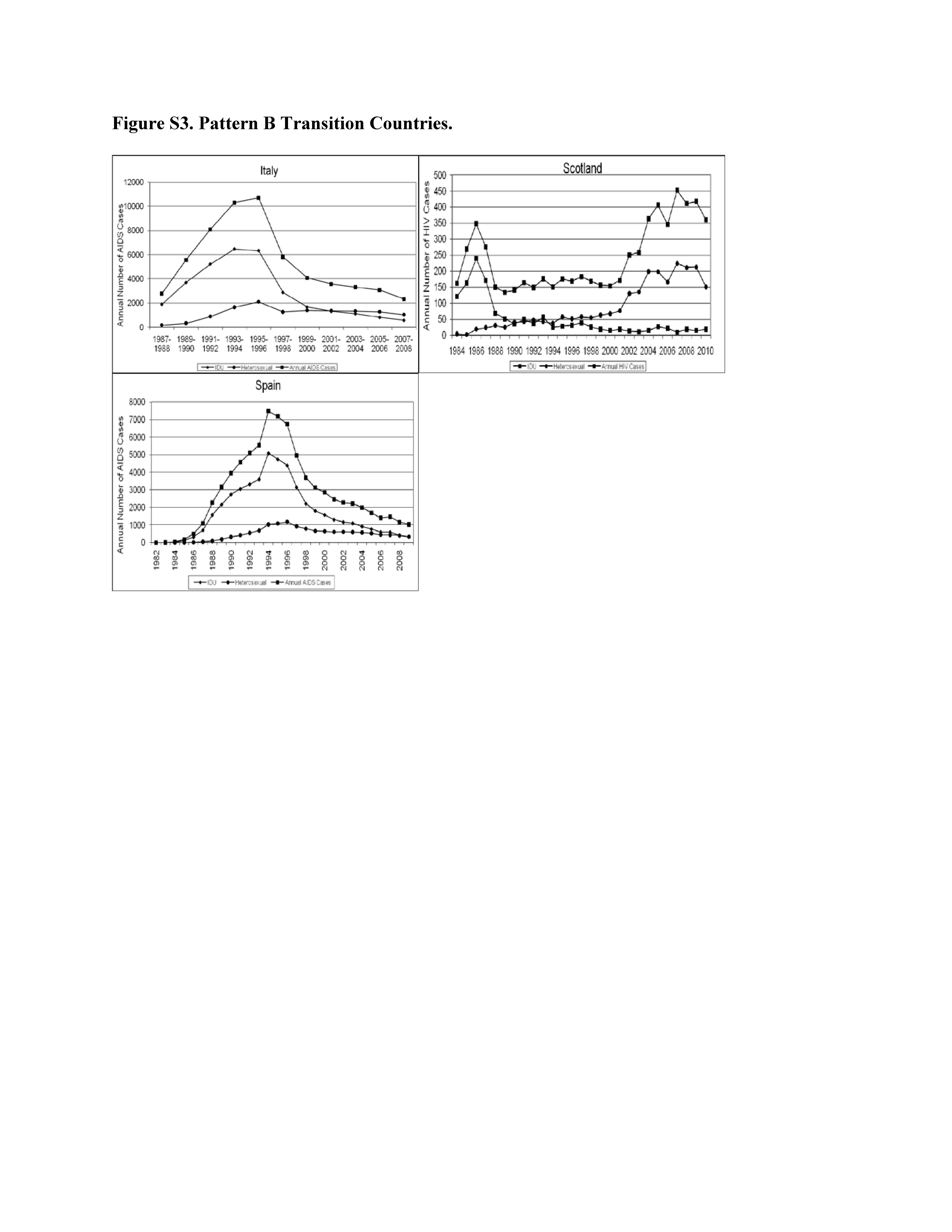

Supplement: Figure S3 — Pattern B transitions from IDU-concentrated to heterosexual epidemics. Locations that experienced transitions from IDU-concentrated to heterosexual sustained HIV/AIDS epidemics and low levels of newly reported HIV/AIDS cases among PWID. Each data point corresponds to the number of newly reported HIV/AIDS cases, by risk group, for each year that surveillance data was available. (TIF) [file pone.0031227.s003.tif]

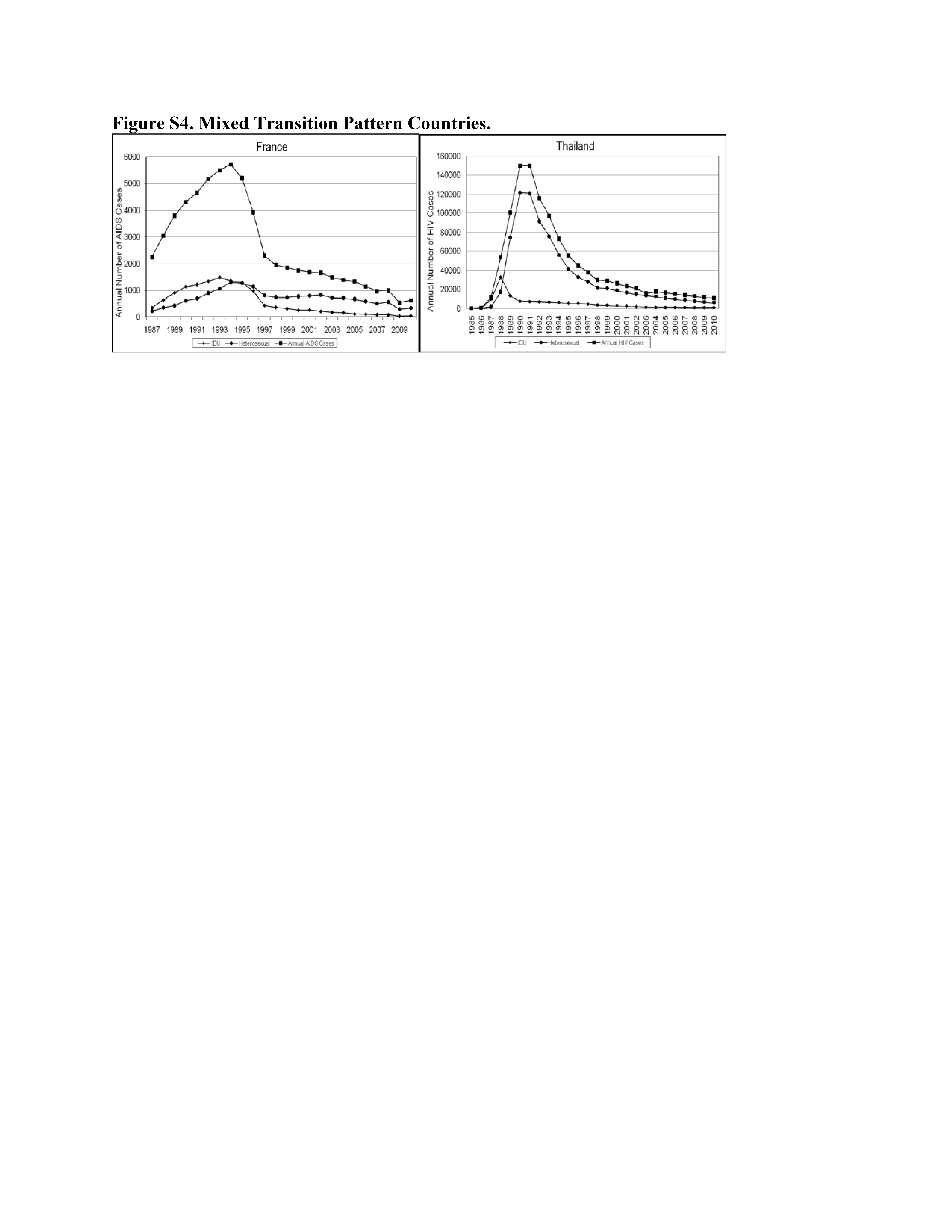

Supplement: Figure S4 — Mixed Transition from IDU-concentrated to heterosexual epidemics. Locations that had experienced a transition from IDU-concentrated to heterosexual sustained HIV/AIDS epidemics, and newly reported HIV/AIDS cases among PWID still surpass newly reported heterosexual HIV/AIDS cases. Each data point corresponds to the number of newly reported HIV/AIDS cases, by risk group, for each year that surveillance data was available. These countries were separated into a separate category due to their unique transitions that did not fit the trends of pattern A or pattern B transitions. (TIF) [file pone.0031227.s004.tif]
